# Outcomes of Radiofrequency Ablation for Dysplastic Barrett's Esophagus: A Comprehensive Review

**DOI:** 10.1155/2016/4249510

**Published:** 2016-12-14

**Authors:** Carmelo Luigiano, Giuseppe Iabichino, Leonardo Henry Eusebi, Monica Arena, Pierluigi Consolo, Carmela Morace, Enrico Opocher, Benedetto Mangiavillano

**Affiliations:** ^1^Unit of Digestive Endoscopy, San Paolo Hospital, Via A. Di Rudiní, No. 8, 20142 Milano, Italy; ^2^HPB Endoscopy, Royal Free Hospital, London, UK; ^3^Department of Medical and Surgical Sciences (DIMEC), University of Bologna, Bologna, Italy; ^4^Department of Medicine and Pharmacology, University of Messina, Hospital “G. Martino”, Via Consolare Valeria, 98124 Messina, Italy; ^5^Department of Surgery, Unit of Hepatobilyopancreatic and Digestive Surgery, San Paolo Hospital, University of Milan, Via A. Di Rudiní, No. 8, 20142 Milano, Italy; ^6^Unit of Digestive Endoscopy, Istituto Clinico Humanitas Mater Domini, Via Gerenzano 2, 21053 Castellanza, Italy

## Abstract

Barrett's esophagus is a condition in which the normal squamous lining of the esophagus has been replaced by columnar epithelium containing intestinal metaplasia induced by recurrent mucosal injury related to gastroesophageal reflux disease. Barrett's esophagus is a premalignant condition that can progress through a dysplasia-carcinoma sequence to esophageal adenocarcinoma. Multiple endoscopic ablative techniques have been developed with the goal of eradicating Barrett's esophagus and preventing neoplastic progression to esophageal adenocarcinoma. For patients with high-grade dysplasia or intramucosal neoplasia, radiofrequency ablation with or without endoscopic resection for visible lesions is currently the most effective and safe treatment available. Recent data demonstrate that, in patients with Barrett's esophagus and low-grade dysplasia confirmed by a second pathologist, ablative therapy results in a statistically significant reduction in progression to high-grade dysplasia and esophageal adenocarcinoma. Treatment of dysplastic Barrett's esophagus with radiofrequency ablation results in complete eradication of both dysplasia and of intestinal metaplasia in a high proportion of patients with a low incidence of adverse events. A high proportion of treated patients maintain the neosquamous epithelium after successful treatment without recurrence of intestinal metaplasia. Following successful endoscopic treatment, endoscopic surveillance should be continued to detect any recurrent intestinal metaplasia and/or dysplasia. This paper reviews all relevant publications on the endoscopic management of Barrett's esophagus using radiofrequency ablation.

## 1. Introduction

Barrett's esophagus (BE) is a premalignant condition in which the normal squamous lining of the esophagus is replaced by columnar epithelium containing intestinal metaplasia (IM), induced by recurrent mucosal injury related to gastroesophageal reflux disease [1].

Patients with BE are at increased risk of developing esophageal adenocarcinoma (EAC), a cancer that has increased nearly sixfold over the last three decades and is associated with a poor, less than 20%, 5-year survival rate [2].

Malignant degeneration of BE is gradual: from nondysplastic IM to low-grade dysplasia (LGD), to high-grade dysplasia (HGD), and eventually progressing to invasive cancer. Nondysplastic BE shows a very low risk of malignant transformation with annual incidence of 0.33%; therefore, endoscopic therapies should not be applied [3].

Recent data demonstrate that, in patients with BE and LGD, ablative therapy results in a significant reduction of progression to HGD or EAC [4, 5].

High-grade dysplasia or intramucosal cancer (IMC) necessitates intervention and endoscopic eradication therapy is the procedure of choice [6].

Endoscopic treatments for BE aim to eliminate Barrett's epithelium, subsequently replaced by normal squamous epithelium. Two distinct endoscopic approaches have been widely used. The first is the complete endoscopic mucosal resection (EMR) of the BE mucosa. The other approach is the ablation of the BE mucosa by using a variety of techniques including photodynamic therapy, argon plasma coagulation (APC), and, more recently, radiofrequency ablation (RFA).

The ablative therapy aims at elimination of BE by inducing superficial tissue necrosis whereby damaged tissue is subsequently replaced by normal squamous mucosa.

Radiofrequency ablation has become the ablative treatment of choice in the management of dysplastic BE [6].

Endoscopic mucosal resection and RFA have both proven to be effective in the eradication of dysplasia and intestinal metaplasia in BE; however, adverse events are significantly more frequent after complete EMR [7].

Radiofrequency is administered by using the Halo system (Barrx Medical, Sunnyvale, Calif), which provides high frequency alternating current to the mucosa with the aim of ablating neoplastic mucosa to allow regrowth of normal squamous mucosa. Reduction in rate of progression to cancer with RFA have been demonstrated in randomized controlled trials in HGD and LGD [4, 8].

However, many patients with HGD or IMC have nodularity in their BE segment and EMR is commonly performed to remove these nodular areas before treatment with RFA [6].

The aim of this review is to summarize results of all important recent publications on this topic to answer relevant clinical questions in order to evaluate the benefit and harm of this therapy.

## 2. Method for Radiofrequency Ablation

This technique requires a specific catheter to apply the radiofrequency ablation to the mucosa. Several sizes of catheters are available, depending on the dimension of the area that needs treating. The Halo 360 (Barrx Medical, Sunnyvale, Calif) is a balloon-based catheter that delivers high-energy pulse to the esophageal lining resulting in a circumferential burn. This system consists of a high power energy generator, a sizing balloon catheter, and balloon-based ablation catheters with different outer diameters. The sizing balloon is used to select the recommended size of the Halo 360. The ablation catheter consists of a noncompliant balloon with a 3 cm long bipolar electrode on its outer surface and available in five outer diameter sizes. Radiofrequency energy is delivered through the electrodes over the balloon resulting in circumferential superficial tissue destruction. The RFA energy is directed uniformly to a depth of 0.5 mm.

The Halo 90, Halo 90 Ultra, and Halo 60 probe catheters may be used to treat focal areas of Barrett's esophagus. These probe catheters are attached to the distal end of the endoscope and can be used to treat selected areas of the mucosa rather than the whole circumference.

Recently, a new device was introduced to eliminate the need for multiple endoscope insertions. Indeed, the new Barrx™ channel RFA endoscopic catheter delivers radiofrequency ablation through the working channel of a flexible endoscope, helping to simplify and reduce procedure time.

## 3. Treatment Efficacy

### 3.1. Low-Grade Dysplasia

There is considerable uncertainty regarding the natural history of LGD within BE. Expert pathology review is important to accurately define patients risk of progression and determining which patients would benefit from treatment. After expert pathology review, 50% to 85% of patients initially diagnosed with LGD may be downstaged to nondysplastic BE with an associated lower risk of neoplastic progression, not requiring treatment [26, 27].

Recent data have shown that, when LGD is confirmed, the annual risk of developing HGD/EAC is 9.1% [28]. A similar high progression rate was seen in the original clinical trial of radiofrequency ablation for dysplastic BE in which 14% of patients in the sham treatment arm developed HGD at one year of follow-up [8].

For patients with a confirmed diagnosis of LGD, RFA is becoming more and more accepted as a therapeutic option to prevent neoplastic progression [6]. Moreover, cost-effectiveness analyses showed that RFA may be both more effective and less expensive compared to surveillance alone for confirmed and stable LGD [29].

The effectiveness of RFA in eradicating dysplasia ranges from 61.5% to 100%, whereas the success of eradication of IM ranges from 61.5% to 91% (Table 1) [4, 5, 8–16]. In particular, with the exception of the study by Okoro et al. that reported lower rates, all other studies reported eradication rates >90% for LGD and >77% for IM. In the AIM Dysplasia Trial, eradication of LGD was achieved in 90.5% of cases in the ablation group at the end of 12 months, and the rate of complete eradication of intestinal metaplasia (CE-IM) was 81% [14].

Sharma et al. evaluated RFA in 39 patients with LGD and achieved complete eradication of dysplasia (CE-D) and CE-IM in 95% and 87% of cases, respectively, with no cases of neoplastic progression, at a median follow-up of 2 years [10].

Phoa et al., in a randomized trial comparing ablation versus surveillance, showed a complete eradication of dysplasia and intestinal metaplasia in 92.6% and 88.2% [4].

A recent multicenter study confirmed that endoscopic ablation has potential benefit over endoscopic surveillance in the management of referred patients with LGD diagnosed by an expert pathologist [5]. This study represents the largest study to date to demonstrate the effectiveness of RFA for LGD in routine practice [5]. The estimated cumulative risk of progression to HGD or EAC within 3 years was 2.9% in the RFA group versus 33.0% in the surveillance group, and mucosal nodularity and multifocal dysplasia were found to be independent predictors for progression to HGD or EAC [5].

Follow-up studies have demonstrated that a high percentage of subjects with LGD maintain CE-D and CE-IM after treatment.

In a follow-up study, Shaheen et al. demonstrated durability of RFA with eradication of LGD and metaplasia in 98% of cases at the end of a 2-year follow-up after ablation therapy [13].

During the follow-up phase of their trial, Phoa et al. reported that CE-D was maintained in 62 out of 63 (98.4%) patients receiving ablation and CE-IM was maintained in 54 out of 60 patients (90.0%) [4].

In a nationwide, multicenter registry of patients treated with RFA, one-fifth of patients, with a mean of 2.4-year follow-up, had recurrent BE and likelihood for recurrence was not influenced by pretreatment dysplasia [30].

### 3.2. High-Grade Intraepithelial/Intramucosal Cancer

Endoscopic therapy is a well-established treatment for more advanced dysplasia, given the high rate of progression to EAC in HGD [31].

The patients with intramucosal cancer can be managed endoscopically given their low risk of local lymph node involvement, whereas patients with lesions invading deep into the submucosa should be considered for esophagectomy [32].

Endoscopic therapy consists in EMR of visible lesions and ablation of any residual BE [6]

Radiofrequency has proven to be very effective also for eliminating BE with HGD and IMC.

The effectiveness of RFA in eradicating HG dysplasia ranges from 74.4% to 100%, and the success of eradication of IM ranges from 41% to 100% (Table 2) [8, 10–25].

In the AIM Dysplasia Trial, CE-D was achieved in 81% of cases in the ablation group at the end of 12 months follow-up, and the rate of CE-IM was 74% [8].

Van Vilsteren et al. showed a complete eradication of dysplasia and metaplasia in 96% (21 out of 22) of patients with BE containing HGD/IMC and visible abnormalities treated with focal EMR followed by RFA [20].

Haidry et al. analyzed data from a large series of patients in the UK who underwent RFA for BE-related neoplasia [21]. Complete eradication of dysplasia was achieved in 81% of the ablation group and CE-IM in 62%, while only 3% of cases developed invasive cancer at 12 months after treatment [21].

Several studies have addressed factors related to unsuccessful RFA treatment in patients with HGD.

A prospective multicenter study demonstrated that poor regression of BE 3 months after the first treatment is a predictor of failure to achieve CE-D/CE-IM, requiring more treatment sessions and a longer treatment period [33].

Another study identified incomplete mucosal healing between treatment sessions as an independent predictor of incomplete eradication of IM [15].

Length of Barrett's segment, uncontrolled acidic reflux, and size of hiatal hernia also seem to be associated with increased failed therapy [16, 34].

Furthermore, recurrence of BE following complete eradication should be considered.

Orman et al. in a systematic review with meta-analysis found an IM recurrence rate of approximately 13%, whereas dysplasia and EAC occurred in 0.9% and 0.7%, respectively, over 1.5 years [35].

Pasricha et al. investigated the rate of recurrence of IM after successful CE-IM in a multicenter registry of patients treated with RFA, and of the 1634 enrolled, 334 (20%) had recurrence of IM and the likelihood for recurrence was associated with increasing age, BE length, and non-Caucasian race [30].

Other studies have reported recurrence rates of up to 33% at 2-year follow-up [21, 36].

Recently, Cotton et al. described the location of biopsies and EMR specimens positive for recurrence during endoscopic surveillance after RFA for BE [37]. Recurrence tended to occur most often around the GEJ; therefore, random biopsies specifically directed around the GEJ have the highest yield [37]. In contrast, recurrence >1 cm proximal to the GEJ was always related to endoscopically visible lesions [37].

An important reason to biopsy the proximal areas of previously treated BE is the possibility of identifying persistence of glandular epithelium beneath the new squamous epithelium, known as buried BE. These buried glands could lead to future neoplastic progression, and the risk of buried BE after ablative therapy is an important concern for all ablative techniques [38].

However, several studies have demonstrated that the presence of buried glands in normal-appearing neosquamous epithelium after RFA is rare [8, 12, 39, 40].

## 4. Comparative Data

Two RCTs of RFA versus only endoscopic surveillance in BE showed that RFA had a high rate of complete eradication of dysplasia and IM and decreased disease progression compared with the control group [4, 8].

In the AIM Dysplasia Trial, 127 patients with dysplastic BE in a 2 : 1 ratio were randomized to receive either RFA (84 patients) or a sham procedure (43 patients) [8]. Among all patients, regardless of the grade of dysplasia, complete eradication of all intestinal metaplasia occurred in 77.4% of the patients assigned to the RFA group, as compared with 2.3% of those assigned to the control group (*P* < 0.001) [8]. Patients who were assigned to the control group were more likely to have disease progression (16.3%) than were those in the RFA group (3.6%, *P* = 0.03) [8]. Among all patients, regardless of the grade of dysplasia, significantly more esophageal cancer cases developed in the control group than in the RFA group (9.3% versus 1.2%, *P* = 0.045) [8].

Phoa et al. in a randomized trial of RFA versus surveillance enrolled 136 patients with a confirmed diagnosis of BE with LGD [4]. Among patients in the RFA group, complete eradication occurred in 92.6% cases of dysplasia and 88.2% of intestinal metaplasia, compared to 27.9% for dysplasia and 0% for intestinal metaplasia among patients in the control group (*P* < 0.001). Complications occurred in 19.1% of patients receiving RFA (*P* < 0.001) [4]. Radiofrequency ablation compared to surveillance reduced the risk of progression to HGD or EAC by 25.0% (1.5% versus 26.5%; 95% CI, 14.1%–35.9%; *P* < 0.001) and the risk of progression to EAC by 7.4% (1.5% versus 8.8%; 95% CI, 0%–14.7%; *P* = 0.03) [4]. Indeed, this study underwent early termination due to superiority of ablation compared to surveillance, since patients in the ablation group were less likely than the control group to progress to high-grade dysplasia or adenocarcinoma [4].

A retrospective study compared 65 BE patients with HGD or IMC treated with EMR and RFA for nodular disease and 104 patients treated with RFA alone for BE with nonnodular disease; EMR and RFA achieved CE-D and CE-IM in 94% and 88% of patients, respectively, compared with 82.7% and 77.6% of patients, respectively, in the RFA alone group (*P* = 0.06 and *P* = 0.13, resp.). The complication rates between the two groups were similar (7.7% versus 9.6%, *P* = 0.79) [41].

Van Vilsteren et al. compared the safety of stepwise radical EMR versus focal EMR followed by RFA for complete eradication of BE containing HGD/IMC [20]. Complete eradication of dysplasia was achieved in 100% (25/25) of patients in the EMR group and in 96% (21/22) of patients in the EMR/RFA group; complete eradication of intestinal metaplasia was achieved in 92% (23/25) of patients in the EMR group and in 96% (21/22) of patients in the EMR/RFA group [20]. However, the rate of posttreatment stenosis was significantly higher in the EMR group (88%) compared with EMR/RFA (*P* < 0.001) [20].

Radiofrequency ablation was also compared with photodynamic therapy (PDT) in a consecutive series of patients with BE [42]. Complete eradication of dysplasia was achieved in 18/33 patients (54.5%) with PDT and in 47/53 patients (88.7%) with RFA (*P* = 0.001) [42]. Comparing the results, PDT was five times more costly than RFA and one patient in the PDT group had an esophageal perforation, managed with nonsurgical measures, while no perforation was seen with RFA group [42].

## 5. Complications

Radiofrequency ablation has a lower complication rate compared with complete EMR [7].

The most common complication associated with esophageal RFA is posttreatment stricture formation with a reported incidence up to 19% [4, 8, 10–16, 21–25].

Chest pain and dysphagia are common but usually resolve spontaneously.

Hemorrhage has also been reported, although less commonly, while esophageal perforations are very rare with a rate of less than 0.6% [43].

Radiofrequency after EMR could result in an increased incidence of stricture formation.

Two studies compared the incidence of complications between patients that underwent RFA after EMR or RFA alone and found no difference in the rate of complications between the two groups [14, 41].

However, a recent meta-analyses found the relative risk for adverse events due to RFA to be about 4-fold higher with EMR than without. The rate of esophageal strictures was higher in the RFA plus EMR group (13.3%) compared to the RFA alone group (5.1%) [43].

## 6. Conclusion

In conclusion, the use of RFA for the treatment of dysplastic BE results in CE-D and CE-IM in a high proportion of patients, with only few recurrences of IM after treatment and low rates of adverse events. Both EMR and RFA have proven to be effective treatments in the eradication of dysplastic BE, although adverse events rates are significantly higher after EMR. Finally, RFA with prior EMR of any nodular areas is the endoscopic treatment of choice for high-grade dysplastic BE.

## Figures and Tables

**Table 1:** Efficacy of radiofrequency ablation for Barrett's esophagus with low-grade dysplasia and intestinal metaplasia.

Author/year	Study design	Setting	Number	BE length	ER before RFA	Efficacy (end of treatment)		Recurrence		Complications
						CE-D (%)	CE-IM (%)	D (%)	IM (%)	
Sharma et al. 2008 [9]	RS	SC	10	4.4 (mean)	0%	90%	90%	0%	0%	10% (1 bleeding)
Sharma et al. 2009 [10]	PS	SC	38	4 (median)	0%	95%×	87%×	NR	NR	2,6% (1 stricture)
Shaheen et al. 2009 [8]	RCT	MC	42	4.6 (mean)	NR	90.5%	81%	—	—	9.5% (5 stricture, 1 bleeding, 2 chest pain)
Pouw et al. 2010 [11]	PS	MC	11	8 (median)	96%	91%	91%	0%	0%	8% (1 bleeding, 1 stricture)^*∗*^
Lyday et al. 2010 [12]	RS	MC	13	3 (median)	0%	100%×	85%	NR	NR	3.9% (9 stricture, 3 bradycardia, 4 bleeding, 1 superficial mucosal injury, 1 fever)^*∗*^
Shaheen et al. 2011 [13]	RCT	MC	52	4.5 (mean)	9%	98%	98%	2%	2%	10.9% (1 bleeding, 3 chest pain, 9 esophageal stricture)^*∗*^
Okoro et al. 2012 [14]	RS	SC	13	4.5 (mean)	0%	61.5%×	61.5%×	NR	NR	16.6% (10 stricture, 5 chest pain)^*∗*^
Bulsiewicz et al. 2013 [15]	RS	SC	44	4 (mean)	9%	93%	86%	NR	NR	8.2% (18 stricture, 2 bleeding)^*∗*^
Dulai et al. 2013 [16]	RS	SC	ULSBE: 5	10.8 (median)	38%^*∗*^	100%	77%^*∗*^	0%	23%^*∗*^	21% (7% postablative mucosal tears and 14% stricture)
			LSBE: 7	4.7 (median)	24%^*∗*^	100%	82%^*∗*^	0%	16%^*∗*^	
Phoa et al. 2014 [4]	RCT	MC	68	4 (mean)	—	92.6%	88.2%	1.6%	10%	19.1% (8 stricture, 3 mucosal laceration)
Small et al. 2015 [5]	RS	MC	45	5 (median)	4.4%	95.6%	77.8%	16%	NR	—

^*∗*^Cumulative data.

BE: Barrett's esophagus; ER: endoscopic mucosal resection; RFA: radiofrequency ablation; RCT: randomized controlled trial; PS: prospective study; RS: retrospective study; SC: single center; MC: multicenter; ULSBE: ultra-long segment Barrett's esophagus; LSBE: long segment Barrett's esophagus; NR: not reported; CE-D: complete eradication of dysplasia; CE-IM: complete eradication of intestinal metaplasia; D: dysplasia; IM: intestinal metaplasia. ×: efficacy at end of follow-up.

**Table 2:** Efficacy of radiofrequency ablation for Barrett's esophagus with high-grade dysplasia/intramucosal carcinoma.

Author/year	Study design	Setting	Number	BE length in cm	ER before RFA	Efficacy (end of treatment)		Recurrence		Complications
						CE-D (%)	CE-IM (%)	D (%)	IM (%)	
Gondrie et al. 2008 [17]	PS	SC	11	7 (median)	58%	91%	100%	0%	0%	9% (1 stricture)
Gondrie et al. 2008 [18]	PS	SC	9	5 (median)	55%	88.9 %	100%	0%	0%	0%
Ganz et al. 2008 [19]	RS	MC	142	6 (median)	17%	80.4%×	54.3%×	NR	NR	0.4 % (1 stricture)
Sharma et al. 2009 [10]	PS	SC	24	6 (median)	8%	79%×	67%×	NR	NR	0%
Shaheen et al. 2009 [8]	RCT	MC	42	5.3 (mean)	NR	81%	74%	—	—	9.5% (5 stricture, 1 bleeding, 2 chest pain)^*∗*^
Pouw et al. 2010 [11]	PS	MC	24	8 (median)	96%	95%	88%	0%	12.5%	8% (1 bleeding, 1 stricture)^*∗*^
Van Vilsteren et al. 2011 [20]	RCT	MC	22	4 (median)	82%	96%	96%	0%	0%	31.8% (3 bleeding, 3 stenosis, 1 acute nontransmural laceration)
Lyday et al. 2010 [12]	RS	MC	10	3 (median)	10%	100%×	80%×	NR	NR	3.9% (9 stricture, 3 bradycardia, 4 bleeding, 1 superficial mucosal injury, 1 fever)^*∗*^
Shaheen et al. 2011 [13]	RCT	MC	54	5.2 (mean)	8%	92.6%	89%	7.4%	11%	10.9% (1 bleeding, 3 chest pain, 9 esophageal stricture)^*∗*^
Okoro et al. 2012 [14]	RS	SC	39 (ER+RFA)	6.9 (mean)	100%	74.4%×	41%×	NR	NR	16.6% (10 stricture, 5 chest pain)^*∗*^
			8 (RFA)	4.5 (mean)	0%	87.5%×	87.5%×			
Bulsiewicz et al. 2013 [15]	RS	SC	166	4 (mean)	39.2%	84.9%	78.3%	NR	NR	8.2% (18 stricture, 2 bleeding)^*∗*^
Dulai et al. 2013 [16]	RS	SC	ULSBE: 25	10.8 (media)	38%^*∗*^	88%	77%^*∗*^	0%	23%	21% (7% postablative mucosal tears and 14% stricture)^*∗*^
			LSBE: 26	4.7 (media)	24%^*∗*^	84%	82%^*∗*^	0%	16%	
Haidry et al. 2013 [21]	RS	MC	335	5.8 (media)	49%	81%	62%	6%	9%	9.3% (30 stricture, 1 perforation)
Strauss et al. 2014 [22]	RS	MC	36	3.5 (mean)	86%	89%	75%	9%	27%	22% (7 stricture, 1 bleeding)
Perry et al. 2014 [23]	RS	SC	17	5 (median)	53%	88%	65%	17.6%	46%	0%
Lada et al. 2014 [24]	RS	SC	57	5.1 (mean)	49%	79%	49%	21%	7.6%	3.8% (2 stricture, 2 chest pain)
Le Page et al. 2015 [25]	RS	SC	36	5 (median)	47.8%	97%	61%	6%	NR	16% (6 chest pain, 1 stricture, 1 bleeding)

^*∗*^Cumulative data.

BE: Barrett's esophagus; ER: endoscopic mucosal resection; RFA: radiofrequency ablation; RCT: randomized controlled trial; PS: prospective study; RS: retrospective study; SC: single center; MC: multicenter; ULSBE: ultra-long segment Barrett's esophagus; LSBE: long segment Barrett's esophagus; NR: not reported; CE-D: complete eradication of dysplasia; CE-IM: complete eradication of intestinal metaplasia; D: dysplasia; IM: intestinal metaplasia. ×: efficacy at end of follow-up.
